# Exploring associations between early substance use and longitudinal socio-occupational functioning in young people engaged in a mental health service

**DOI:** 10.1371/journal.pone.0210877

**Published:** 2019-01-17

**Authors:** Jacob J. Crouse, Kate M. Chitty, Frank Iorfino, Django White, Alissa Nichles, Natalia Zmicerevska, Adam J. Guastella, Ahmed A. Moustafa, Daniel F. Hermens, Elizabeth M. Scott, Ian B. Hickie

**Affiliations:** 1 Youth Mental Health Team, Brain and Mind Centre, The University of Sydney, Camperdown, NSW, Australia; 2 Translational Australian Clinical Toxicology (TACT) Research Group, Discipline of Pharmacology, The University of Sydney, Camperdown, NSW, Australia; 3 School of Social Sciences and Psychology, Western Sydney University, Milperra, NSW, Australia; 4 MARCS Institute for Brain, Behaviour and Development, Milperra, NSW, Australia; 5 Department of Social Sciences, College of Arts and Sciences, Qatar University, Doha, Qatar; 6 Sunshine Coast Mind and Neuroscience—Thompson Institute, University of the Sunshine Coast, Birtinya, QLD, Australia; 7 Notre Dame Medical School, Sydney, NSW, Australia; University Hospital Carl Gustav Carus, Technische Universitat Dresden, GERMANY

## Abstract

Neuropsychiatric disorders (including substance misuse) are associated with the greatest burden of functional disability in young people, and contributory factors remain poorly understood. Early-onset substance use is one candidate risk factor which may inform functional prognosis and facilitate direction of interventions aiming to curtail impairment. Accordingly, we modelled associations between early-onset use of alcohol, tobacco, cannabis and amphetamine-type stimulants (ATSs) and longitudinal socio-occupational functioning (indexed by the Social and Occupational Functioning Assessment Scale) in an observational cohort presenting to early intervention mental health services. A clinical proforma collated demographic, clinical, and socio-occupational information for up to 60-months from presentation to services in young people aged 17–30. Of the wider cohort (n = 2398), 446 participants were selected with complete alcohol and substance use data. Latent class analysis was used to derive an ‘early-onset’ (n = 243) and ‘later-onset’ class (n = 203) based on age of first use of alcohol, tobacco, cannabis and ATSs. Maximum-likelihood multilevel analyses modelled functioning over time in care and tested associations with substance use latent class, age, gender and diagnosis. Membership in the ‘early-onset’ class (B = -1.64, p = 0.05), male gender (B = -3.27, *p*<0.001) and psychotic disorder diagnosis (B = -7.62, *p*<0.001) were associated with poorer functioning at presentation and at least one other time-point. To our knowledge, this is the first study to explore associations of early-onset substance use and longitudinal functioning in a cohort of young people with mental disorders. The identified factors may be useful for directing specific social (e.g. Social Recovery Therapy) or occupational (e.g. Individual Placement and Support) interventions to at-risk individuals, early in illness course.

## Introduction

The emergence of a mental disorder during adolescence or early adulthood may profoundly and pervasively impact a young person’s educational achievement, workforce participation and social engagement [1–3]. Neuropsychiatric disorders (including substance misuse) are the greatest cause of years lived with disability for young people aged 10–24 [4], and disability-adjusted life years associated with common mental disorders (e.g. depression and anxiety) reach their peak between 10–29 years of age [5]. The reasons for this high burden of disability are complex, involving a coalescence of factors operating within a formative and sensitive phase of social, cognitive and neurobiological development [6–9]. Importantly, strong evidence from longitudinal cohort studies suggests that functional impairment is both a cause and a consequence of mental ill-health [2, 3, 10–19], underscoring the need to consider both domains in assessment and treatment. In keeping with these observations, there has been a gradual shift toward more holistic models of recovery which take into account an individual’s ability to adaptively and meaningfully participate in work and social relationships [20, 21]. This shift complements patient reports citing loneliness, social isolation, financial problems and unemployment as their top-ranked challenges, above symptoms [22]. Attending to functional impairment in young people is especially important, as efforts made early in the course of illness (when trajectories are most malleable) are more likely to be impactful [23, 24]. Accordingly, there is a critical need for identification of factors driving impairment in the early phases of mental disorders in order to direct interventions to at-risk individuals.

Indeed, functional impairment is common and substantial at presentation to early intervention mental health services across a wide array of anxious, psychotic and mood syndromes [25–30]. A recent report from our group described multiple empirical trajectories of functioning over time in care, with substantial variability in improvement, decline and stability among young patients [31]. While some factors associated with poor functioning in psychiatric cohorts have been identified, including male gender, younger age, suicidality, cognitive impairment, substance and illness comorbidity, and greater illness stage [25, 27, 28, 31–34], considerable variance remains unaccounted for. One candidate factor that has received little attention in youth mental health cohorts is early-onset substance use.

In general populations (e.g. school-, birth- and population-based cohorts), it is well-established that early-onset use of alcohol, tobacco, cannabis and amphetamine-type stimulants (ATSs) is associated with numerous poor outcomes. For instance, early-onset alcohol use (i.e. before age 15) is associated with increased risk for future alcohol-related problems and substance dependence, academic difficulties, and employment problems in early adulthood [35–38]. Early-onset tobacco use (i.e. before age 15) predicts persistent cigarette smoking and dependence, school-dropout and psychiatric morbidity, with adolescent-initiators who continue smoking into adulthood at especially high-risk of negative outcomes [39–44]. Early-onset cannabis use (i.e. before age 16) is related to an increased risk for psychosis, cannabis dependence, school-dropout, unemployment at age 18 and socio-occupational difficulties at age 25 [45–48]. Finally, data describing outcomes associated with early-onset ATS use (e.g. methamphetamine, cocaine, MDMA) is scarce, however, some work suggests that early-onset methamphetamine use increases risk for psychosis, dependence and criminal activity [49–51], and early-onset cocaine use is associated with greater legal and psychiatric problems [52, 53]. Unfortunately, the above research has largely been restricted to general population samples, limiting generalisability to treatment-seeking young people with common mental disorders.

As there is no agreed upon cut-point for early- versus later-onset substance use and a range of ages reported in the literature, we chose to empirically derive latent classes of substance users as a function of their age of first use across our four substances of interest (alcohol, tobacco, cannabis and ATSs). Our first aim was to determine whether an earlier-onset substance class was associated with poorer longitudinal socio-occupational functioning (up to five years) in an observational cohort of young people accessing early intervention mental health services in Sydney, Australia. As a secondary question, we aimed to test a putative *developmental-psychosis* typology of mental disorders [54] with respect to functioning and substance use. Specifically, would individuals with a neurodevelopmental or psychotic disorder have poorer longitudinal functioning relative to their peers without either disorder, and, would participants with a neurodevelopmental or psychotic disorder who also reported earlier-onset substance use have even poorer functioning?

We hypothesised that: (i) the latent class with the earliest onset of substance use across alcohol, tobacco, cannabis and ATSs would be associated with lower functioning at presentation relative to the other class(s); (ii) a diagnosed neurodevelopmental or psychotic disorder would be associated with lower functioning at presentation and longitudinally; (iii) younger age would be associated with lower functioning at presentation; and (iv) male gender would be associated with poorer functioning at presentation. An additional exploratory question was whether the earlier-onset class would be associated with poorer functioning over time in contact with clinical services.

## Methods

### Human ethics

This study and the consent procedure were approved by the University of Sydney Human Ethics Committee (Project numbers: 2012/1626 and 2012/1631) and conducted in accordance with the Declaration of Helsinki. Written informed consent was obtained from participants aged 16 years and older, and parental/guardian consent was obtained for participants younger than 16 years.

### Participants

Participants were drawn from a naturalistic, longitudinal cohort of young people, the *‘Brain and Mind Centre Optymise Cohort’* (n = 2398, mean age 18.8 ± 3.8 years, 58.7% female), who were accessing ‘*headspace’* and associated early intervention mental health clinics in Sydney, Australia. *headspace* is Australia’s youth mental health initiative, which aims to provide youth-friendly and highly-accessible early intervention services for young people with emerging mental and substance use disorders [55, 56]. Primarily attracting young people with a wide range of mental health problems (typically anxiety, mood and/or psychotic syndromes), *headspace* consists of an integrated mixed of primary-level services and more specialised services (e.g. psychiatric, drug and alcohol, occupational support).

With informed consent, study participants were recruited to a case register for mood, psychotic, developmental and other mental disorders between January 2005 and January 2018. All participants were receiving ongoing clinician-based case management and relevant psychosocial and/or medical interventions throughout the duration of care, which may have involved contact with a psychiatrist, psychologist, occupational therapist, support worker, or hospitalisation for those whose need exceeded the capacity of the primary care services.

Individuals were included in the present study if they met the following criteria: (i) aged 17–30 years at the time of initial assessment (T1); and (ii) had completed the World Health Organization’s ‘*Alcohol*, *Substance and Smoking Involvement Screening Test*, *Version 2’* (WHO-ASSIST-2). We added a further question to item 1 of the WHO-ASSIST-2 (lifetime use) to collect age of first use data: “*If yes*, *at what age did you first use*?*”*. Exclusion criteria included: (i) medical instability or lack of capacity to provide informed consent (determined by a treating psychiatrist); (ii) medical illness with cognitive sequelae (e.g. epilepsy, cancer); (iii) clinically-evident intellectual disability; and/or (iv) insufficient English-language ability. Of the wider *Optymise* cohort, 446 participants were included in analyses (see Participant Flow Diagram in S1 Fig).

### Data collection

With consent, trained research psychologists and medical officers conducted a medical file audit to collate demographic, clinical and socio-occupational information at pre-specified intervals utilizing a specifically designed clinical proforma. These methods have been described previously in studies examining trajectories of functioning and suicidality [31, 34]. The earliest available comprehensive assessment at the service was represented as the initial timepoint (T1) for each participant, with T1 date determining the follow-up timepoints: 3-months (T2), 6-months (T3), 12-months (T4), 2-years (T5), 3-years (T6), 4-years (T7), and 5-years (T8). A “time-last-seen” entry was also recorded; however, this was not included in the current study. If no clinical notes were available within ±1-month of the 3- and 6-month timepoints, or ± 3-months of the remaining timepoints (T4-T8), then this particular entry was omitted. When data were available for a specified timepoint, all clinical notes collected after the preceding entry, up to and including the current entry, were used to complete the form.

### Clinical proforma

The clinical proforma captures key information about the current presentation and specific illness course characteristics, with an earlier iteration previously reported [27, 57]. The proforma collects information regarding: (i) demographics; (ii) mental health diagnoses (based on Diagnostic and Statistical Manual of Mental Disorders (DSM-5) criteria); (iii) clinical course (e.g. clinical stage, hospitalizations, childhood diagnoses); (iv) comorbidities (e.g. physical health problems, suicidal thoughts/behaviours); (v) and socio-occupational functioning, assessed using the Social and Occupational Functioning Assessment Scale (SOFAS), which is the outcome variable in this study. The SOFAS is a clinician-rated 100-point scale used to assess an individual’s level of social and occupational functioning along a continuum ranging from optimum functioning to important functional impairment (lower scores indicating poorer functioning). The SOFAS has been reported to have good construct validity (e.g. strong correlations with patient-reported difficulties in interpersonal relations and social adjustment [58]), excellent inter-rater reliability (i.e. intraclass correlation coefficient [ICC] > 0.74 [58]), and predictive validity (e.g. for length of initial psychiatric inpatient stay and two-year outcome [59]).

As we aimed to test a developmental-psychosis trajectory [54] hypothesis, participants were dichotomously coded at T1 with either the presence (1) or absence (0) of a psychotic disorder (including DSM-5 schizophrenia [n = 20]; schizoaffective disorder [n = 6]; substance/medication induced psychotic disorder [n = 7]; brief psychotic disorder [n = 6]; schizophreniform disorder [n = 3]; and psychotic disorder not otherwise specified [n = 8]) and presence (1) or absence (0) of a neurodevelopmental disorder (including DSM-5 autism-spectrum disorder [n = 8] and attention-deficit/hyperactivity disorder [n = 8]). Diagnoses were specified according to DSM-5 criteria [60], however, due to differences in the timing of presentation to clinical services clinical notes may have been based on previous iterations of the DSM.

### Statistical analyses

Using the statistical program ‘Mplus’ [61], we conducted latent class (or latent profile) analyses (LCA) to derive empirical classes of substance users, with participants’ age of first use of alcohol, tobacco, cannabis and ATSs representing the input variables. As Mplus uses full-maximum-likelihood estimation to make use of all available data [62–64], participants with no lifetime use for a particular substance (and therefore no age of first use for that substance) were included in analyses. LCAs were run for 1–5 classes, with ample random starts and iterations used to arrive at a replicable best solution for each given number of classes (which was confirmed by a large number of replicated loglikelihoods for each model). Our choice of the number of classes that had a good balance of model fit and parsimony was informed by running 100 parametric bootstraps and comparing likelihood ratio test statistics, as well as inspecting the number of boundary conditions for each number of classes. Membership in a latent class was then dummy-coded (e.g. 1 = ‘*member of class 1*’; 0 = ‘*not a member of class 1’*) and used as a predictor variable in the next step of multilevel modelling.

Multilevel analyses were conducted using the ‘nlme’ package [65] for the statistical programming language *R* (version 3.4.2), utilizing full-maximum-likelihood estimation. This method represents a powerful way to assess change in a continuous dimension (e.g. SOFAS) longitudinally and within-participants, circumventing limitations associated with alternative repeated-measures techniques. Advantages of this method include: (i) tolerance of unbalanced assessment intervals; (ii) inclusion of participants with missing follow-up data (i.e. no list-wise deletion for missing timepoints); and (iii) does not assume independence of observations (which is unlikely to be met for within-participant repeated-measure data).

Our analyses were conducted sequentially. First, we constructed an unconditional model (i.e. no predictors) positing a linear change trajectory in SOFAS without attempting to predict inter-individual variation in parameters by between-subject factors. We additionally tested whether a non-linear term would provide a superior fit (as functional change is likely dynamic over time). Next, we fit a continuous autoregressive covariance structure, as we expected greater correlation in SOFAS scores at nearer timepoints than farther timepoints. We proceeded in conducting a set of conditional analyses examining systematic inter-individual differences in intercept and slope as a function of several pre-determined demographic and diagnostic factors (fixed effects), with the initial order entry substantively informed by the literature.

Normality of residuals was visually inspected using Q-Q plots, with an approximate normal distribution evident. Multicollinearity between predictors was assessed using the variation inflation factor (VIF), with no predictor variables observed to have a VIF exceeding 2.0. Model coefficients (B) are presented alongside standard errors, 95% confidence intervals (CI) and parameter-specific *p*-values. Deviance statistics are provided for each model, including the Akaike information criterion (AIC), Bayesian information criterion (BIC) and the Log-Likelihood. Goodness-of-fit between models was compared using the likelihood ratio test (LRT) statistic (which expresses how many times more likely the data are under one model relative to another) and *p*-values, with α level set at 0.05.

## Results

### Sample demographics and clinical characteristics

At T1, the included sample comprised four-hundred-and-forty-six young people (aged 17–30; *M* = 21.2; *SD* = 3.2), with 55.6% female gender. Presenting diagnoses, age of first use information, and sample size at each time-point are reported in Table 1. Baseline demographics of participants lost to follow up over 60-months are presented in Table 2.

**Table 1:** Demographic, age of substance use onset and presenting clinical diagnostic information (*n* = 446).

	*M* ± *SD* or *N* (%)
**Demographics**	
Gender (female)	248 (55.6)
Age at entry	21.2 ± 3.2
**Substance use onset (age, yrs)**	
*Alcohol*	15.1 ± 2.4
*Tobacco*	15.6 ± 2.9
*Cannabis*	16.2 ± 2.6
*Amphetamine-type stimulant*	17.9 ± 2.6
**Presenting clinical diagnosis**	
Depressive disorder	202 (45.3)
Bipolar disorder	69 (15.5)
Anxiety disorder	77 (17.3)
Psychotic disorder	50 (11.2)
Neurodevelopmental disorder	16 (3.6)
Substance or addictive disorder	8 (1.8)
Other	22 (4.9)
No diagnosis	2 (0.4)
**Available timepoints**	
T1 (Entry)	446 (100.0)
T2 (3-months)	275 (61.7)
T3 (6-months)	238 (53.4)
T4 (12-months)	218 (48.9)
T5 (2-years)	172 (38.6)
T6 (3-years)	128 (28.7)
T7 (4-years)	97 (21.7)
T8 (5-years)	56 (12.6)

**Table 2:** Baseline demographics of participants lost to follow-up over 5 years (*n* = 446).

	Final timepoint with available data for each participant							
	T1 (n = 72)	T2 (n = 40)	T3 (n = 55)	T4 (n = 65)	T5 (n = 63)	T6 (n = 46)	T7 (n = 49)	T8 (n = 56)
**Age at entry**	22.8 ± 3.3	20.7 ± 3.2	21.5 ± 3.1	21.0 ± 3.3	21.1 ± 3.3	20.9 ± 3.3	20.7 ± 2.6	20.4 ± 2.6
**Gender (female)**	33 (46%)	20 (50%)	30 (55%)	42 (65%)	28 (44%)	25 (54%)	34 (69%)	35 (63%)
**T1 SOFAS**	59.5 ± 13.6	61.2 ± 9.7	61.9 ± 8.7	58.8 ± 9.2	63.3 ± 9.0	58.4 ± 6.7	60.6 ± 9.0	60.2 ± 9.0
**T1 Psychotic dx**	23 (32%)	3 (8%)	5 (9%)	4 (6%)	5 (8%)	3 (7%)	3 (6%)	4 (7%)
**T1 ND dx**	3 (4%)	4 (10%)	0 (0%)	2 (3%)	2 (3%)	3 (7%)	2 (4%)	0 (0%)
**Alcohol AFU**	14.9 ± 2.5	15.4 ± 1.9	15.5 ± 2.2	15.1 ± 2.1	15.3 ± 2.4	14.4 ± 2.4	15.0 ± 2.5	15.1 ± 2.9
**Tobacco AFU**	15.9 ± 3.2	16.0 ± 2.3	14.8 ± 2.8	16.0 ± 2.6	16.3 ± 3.3	14.7 ± 2.5	15.1 ± 2.7	15.3 ± 2.7
**Cannabis AFU**	16.3 ± 3.0	16.2 ± 2.1	16.5 ± 2.6	16.3 ± 2.6	16.9 ± 2.6	15.1 ± 2.2	15.7 ± 2.0	16.4 ± 3.2
**ATS AFU**	18.1 ± 2.9	17.3 ± 2.7	18.3 ± 2.5	17.7 ± 2.7	18.3 ± 2.7	17.6 ± 2.6	17.6 ± 2.3	17.8 ± 2.5

T1 = service entry; T2 = 3-months; T3 = 6-months; T4 = 1-year; T5 = 2-years; T6 = 3-years; T7 = 4-years; T8 = 5-years; Psychotic dx = psychotic disorder; ND dx = neurodevelopmental diagnosis; ATS = amphetamine-type stimulant; AFU = age of first use

### Latent class analyses

Analyses were run for 1–5 classes in order to arrive at the optimal number of classes representing the data. Information criteria and 100 parametric bootstrapped likelihood ratio tests (LRTs) were used to guide the decision of the number of classes. A sufficient number of random starts and iterations were used to arrive at a replicable solution, which was confirmed by a large number of replicated loglikelihoods for each model. All model estimations terminated normally.

A 3-class solution was found to be the best-fitting model with respect to the information criteria, LRT statistics (see Tables 3 and 4) and parsimony. However, a 2-class solution also provided a good fit to the data, comprised fewer boundary conditions than the 3-class solution, had an adequate sample size in each class to meaningfully model in longitudinal analyses, and was a more parsimonious solution with respect to our research question (i.e. early-onset versus later-onset substance users). We accordingly settled on a 2-class solution, which described an early-onset (n = 243) and later-onset (n = 203) substance use class (see Table 5 for class descriptives).

**Table 3:** Information criteria for 1–5 latent class estimations.

	AIC	BIC	Sample-size adjusted BIC	Entropy
Number of latent classes				
1	6089.80	6122.60	6097.21	-
2	5842.20	5895.50	5854.25	0.62
3	5707.71	5781.52	5724.40	0.71
4	5633.35	5727.66	5654.67	0.72
5	5583.08	5697.89	5609.03	0.75

AIC = Akaike Information Criterion; BIC = Bayesian Information Criterion

**Table 4:** Model comparisons for 5 latent class estimations using 100 parametric bootstrapped likelihood ratio tests.

	Parametric boostrapped likelihood ratio test (2 times the Loglikelihood difference)	p
Number of latent classes		
2 versus 1	257.60	<0.001
3 versus 2	144.49	<0.001
4 versus 3	84.36	<0.001
5 versus 4	60.27	<0.001

**Table 5:** Characteristics of early-onset and later-onset substance use latent classes.

	Latent class 1 *Early-onset* *(n = 243)*	Latent class 2 *Later-onset* *(n = 203)*
	*M* ± *SD* or *N* (%)	
Age at entry	21.1 ± 3.3	21.4 ± 3.1
Gender (female)	129 (53%)	119 (59%)
Substance use onset (age, years)		
Alcohol	13.6 ± 1.9	16.9 ± 1.5
Tobacco	14.0 ± 1.8	18.3 ± 2.2
Cannabis	15.0 ± 1.9	18.7 ± 2.2
Amphetamine-type stimulant	17.0 ± 2.3	20.1 ± 2.0

### Multilevel modelling: Unconditional analyses

Next, we began constructing our multilevel models by specifying an unconditional model (i.e. no predictors) with random intercepts. We then modelled the fixed relationship between SOFAS and ‘time’ with a linear term, which was significant and indicated a positive slope in SOFAS change over time across the sample (B = 0.31, p<0.001). We tested whether a quadratic trend in ‘time’ was a superior fit to the data, which was non-significant (*p* = 0.68) and did not improve model fit (LRT = 0.17, *p* = 0.68), indicating that a linear trend was appropriate. Next, slopes were randomly varied across participants, which is intuitive in that individuals are likely to be variable in their rate of improvement, decline or stability over time. The random slopes and random intercept model fit the data substantially better than the fixed slopes model (LRT = 111.21, *p*<0.001). We next determined whether there was autocorrelation in SOFAS scores across timepoints by fitting an autoregressive covariance structure to the data, which improved model fit (LRT = 27.99, *p*<0.001).

### Multilevel modelling: Conditional analyses

We then examined factors that might explain intercept variation. We first entered the presence of a psychotic disorder at presentation to the model, which was significant (B = -7.74, *p*<0.001) and improved fit (LRT = 30.77, *p*<0.001). Next, the presence of a neurodevelopmental disorder was added, which was neither significant at our a priori alpha of 0.05 (B = -4.19, *p* = 0.07) nor improved fit (LRT = 3.39, *p* = 0.07), and was therefore excluded from further modelling. We then added gender to the model, which was significant (male gender; B = -3.34, *p*<0.001) and improved model fit (LRT = 14.97, *p*<0.001), followed by age at each time-point which was non-significant (B = -0.01, *p* = 0.92), did not improve fit (LRT = 0.01, *p* = 0.92), and was not included in further modelling. We next added membership in the ‘early-onset’ latent class (with the ‘later-onset’ class serving as reference) to the model, which was significant (B = -1.65, p = 0.05) and improved model fit (LRT = 3.94, p = 0.05). There was no significant interaction between membership in the early-onset class and having a psychotic disorder (B = -1.24, p = 0.65).

**Fig 1:**
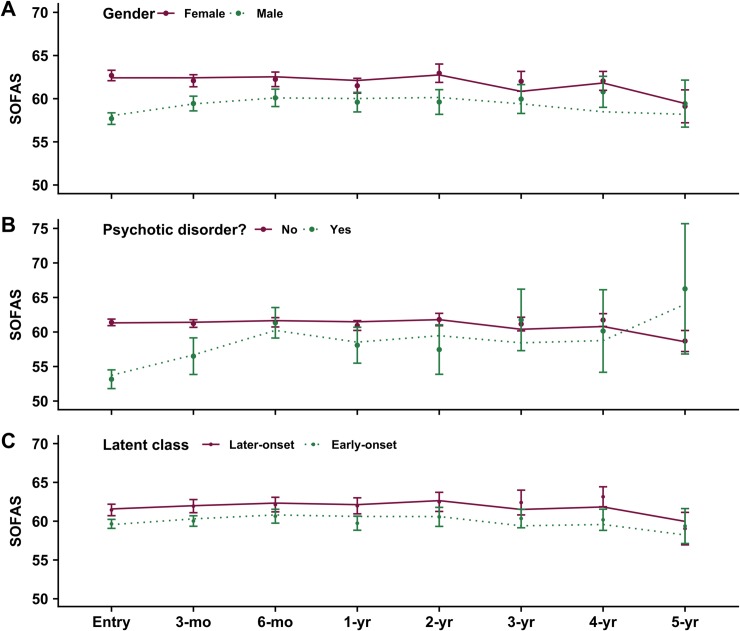
Observed data (± SE) and linear model fits for socio-occupational functioning (SOFAS) over 5-years in 446 young people with common mental disorders. Note: filled circles = mean observed data; bars = standard error; lines = fitted model.

Finally, we tested whether statistical interactions between predictor variables would be associated with variability in the rate of SOFAS change over time (i.e. slope). We observed a trend towards a significant ‘time’ by gender interaction (male gender; B = 0.44, *p* = 0.06), and a trend toward improved model fit (LRT = 3.51, *p* = 0.06), which would indicate that males had a greater rate of SOFAS improvement over time than females. There were no significant interactions between ‘time’ and the ‘early-onset’ latent class (B = -0.17, *p* = 0.46) or ‘time’ and psychotic disorder (B = 0.61, *p* = 0.17). Final model coefficients are presented in Table 6, and fitted models are plotted in Fig 1.

**Table 6:** Final linear multilevel model (n = 446).

Predictor	Model		
Fixed effects	Coefficients (95% CI)	t	*p*
Intercept	63.67 (62.26, 65.07) ***	89.01	<0.001
Time	0.13 (-0.16, 0.42)	0.88	.378
Psychotic disorder	-6.41 (-9.12, -3.70) ***	-4.64	<0.001
Gender (male)	-3.69 (-5.42, -1.96) ***	-4.18	<0.001
‘Early-onset’ class	-1.66 (-3.28, -0.03) *	-2.00	.046
**Interactions**			
Time x Gender	0.44 (-0.02, 0.89)	1.88	.061
**Random effects**	**SD**		
Intercept	7.29		
Time	1.17		
Residual	5.85		
**Deviance statistics**			
AIC	11092.95		
BIC	11152.27		
logLik	-5535.47		

* p<0.05

** p<0.01

*** p<0.001

AIC = Akaike Information Criterion; BIC = Bayesian Information Criterion; logLik = loglikelihood

## Discussion

Functional impairment is common and often pervasive in young people with mental health problems [31] and identification of factors predictive of longitudinal functioning is warranted in order to inform clinical prognosis and facilitate treatment selection. The present study sought to explore several candidate predictive factors of functioning at service entry and over time in contact with clinical services, observing that: i) membership in a latent class of early-onset substance users was associated with lower functioning at service entry and 3-, 12- and 48-months later (see Fig 1C); ii) male gender was associated with lower functioning throughout the first 6-months of care and at 2-years after service entry (see Fig 1A); and iii) a psychotic disorder at service entry was associated with lower functioning throughout the first 3-months in care (see Fig 1B). Against expectations, neither age nor having a neurodevelopmental disorder were associated with poorer functioning.

Our finding of poorer functioning among early-onset substance users may have several explanations. First, it is possible that early- and later-onset substance users may be neurocognitively or neurobiologically distinct, with differences mapping onto differential capacities for functioning. While a number of preclinical and human studies have revealed neurocognitive and neurobiological changes associated with heavy alcohol use during adolescence [66–69], few have investigated the effects of age of initiation. One recent preliminary study however reported associations between poorer processing speed and visual attention with earlier age of first drink, and poorer cognitive inhibition and working memory with earlier age of weekly drinking onset [70]. Importantly, these effects were robust to controlling for baseline neurocognition, severity of substance use and several family and social environment factors [70]. With respect to tobacco, a number of preclinical and human studies have suggested a neurotoxic effect of early exposure to nicotine (during adolescence) on brain and neurocognitive development [71–73]. Work in animal models has demonstrated long-lasting deficits in attention following administration of nicotine during adolescence [74], with lasting synaptic changes to dopaminergic and glutamatergic signalling in prefrontal cortex thought to represent two mechanisms underpinning attentional deficits [74, 75]. In humans, earlier initiation of tobacco smoking has been associated with deficits in response inhibition [76], sustained attention [76], and working memory [71]. Likewise, earlier use of cannabis during adolescence has been associated with poorer performance on a number of cognitive tasks indexing decision-making [77], verbal IQ [78], impulsivity [79], executive functions [80, 81] and memory [82], with suggestions that cannabis use during adolescence may perturb developmental processes such as white matter development and synaptic pruning [83]. Importantly, many of these studies are cross-sectional and collect retrospective age of onset data, and there is a need for prospective and longitudinal studies tracking adolescents before and after initiation of substance use to clarify the links between brain health and adolescent substance use [84, 85]. An alternative explanation may be that antecedent factors preceding substance use initiation may differentiate early- and later-onset users, which may signal shared liability for both early substance involvement and socio-occupational problems. For instance, Ellickson and colleagues [38] observed in a school-based cohort that early-onset and experimental drinkers were more likely than non-drinkers to have academic problems in school and employment problems in early adulthood, suggesting that early drinkers may not ‘mature’ out of problematic antecedent lifestyles that may represent shared risk for early and later difficulties. Other antecedent factors may include: i) early-onset mental health problems [86–88]; ii) socio-economic and family-level factors, including disrupted family structures, substance-misusing parents and siblings, social disadvantage, trauma-exposure, and poor parental monitoring and parent-child relationships [37, 89–94]; or iii) personality and behavioral factors, such as male gender, teacher-reported aggressive behaviour, conduct symptoms, positive alcohol expectancies, and reward-related personality traits [37, 95–99]. On balance, early-onset substance use may represent an associative (rather than causal) marker for the above confounding factors which may in turn increase risk for functional problems.

Based on a putative typology of adolescent-onset mental disorders [54], we hypothesised that early substance users who also had a neurodevelopmental or psychotic disorder (i.e. a *developmental-psychosis* trajectory) would be at risk of poorer outcome. While main effects of early-onset substance use and psychotic disorder on functioning were evident, we did not observe a statistical interaction between them. Nevertheless, the clustering of male gender, early-onset substance use and psychosis with poor functioning is congruent with this putative typology [54], and warrants further examination with modelling of larger samples enriched with these factors.

Finally, male gender was associated with lower functioning across the first 6-months of care and at 2-years (Fig 1A). This dovetails with the wider literature and may result from greater impairment prior to illness-onset or help-seeking due to other risk factors (e.g. neurodevelopmental or cognitive risk factors more common in boys), delayed help-seeking behaviour associated with poor health literacy [100], or the lack of development of suitable healthcare environments engaging to young men [101].

There are several limitations and potential sources of bias in this study. First, the SOFAS indexes both social and occupational functioning within one scale, which while useful in characterising the ‘gestalt’ of the individual’s circumstances may also obfuscate specific strengths and weaknesses. Second, age of first substance use was self-reported and may suffer from recall bias or related inaccuracies. Moreover, our sample was biased toward young people engaged in help-seeking behaviour and may not be generalizable to individuals who do not seek help or enter clinical services due to poor insight, low support, or other factors. Finally, loss to follow-up within this subset of the wider cohort may have biased model estimates. However, characteristics presented in Table 2 suggest no substantial differences in T1 SOFAS, gender distribution or T1 age across participants with differing final timepoints with available data. With these limitations in mind, we recommend replication in a similar youth mental health cohort.

In sum, our work highlights a substantial need for enhanced socio-occupational intervention and assistance in young people with mental ill-health, especially as early disengagement may herald protracted problems. In a subset of our larger cohort, we show that early-onset substance use is associated with poorer functioning at service entry and at several time-points throughout care, highlighting an at-risk group which may benefit from additional social and occupational treatment and support (e.g. Individual Placement and Support, Social Recovery Therapy [102, 103]).

## Supporting information

S1 Fig Participant flow diagram. (TIF)

S1 File Study data. (CSV)
