# Supplementary figures and images for: Exploring associations between early substance use and longitudinal socio-occupational functioning in young people engaged in a mental health service
Source: PLoS One. 2019 Jan 17;14(1):e0210877. doi: 10.1371/journal.pone.0210877 (PMC6336340; doi:10.1371/journal.pone.0210877)

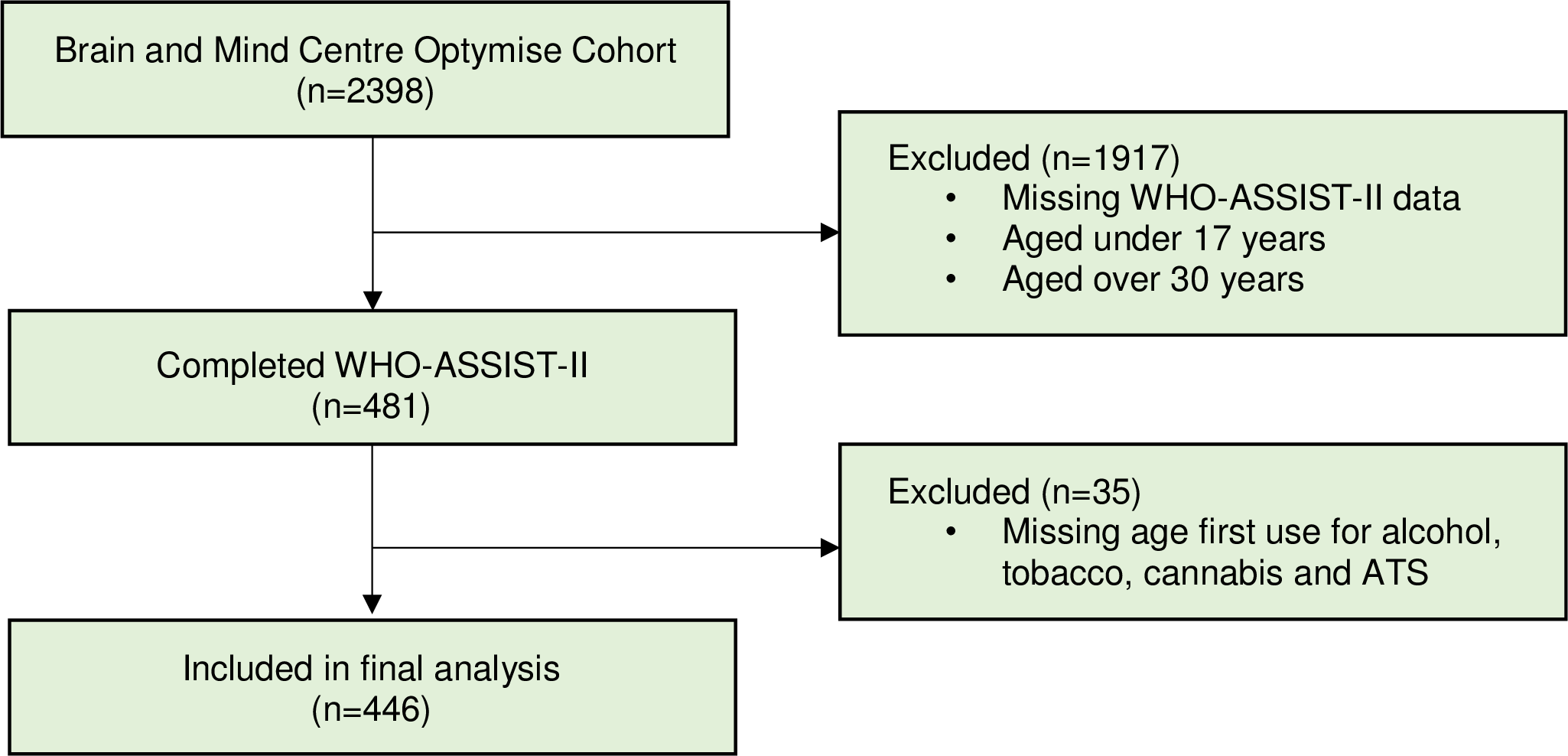

Supplement: S1 Fig — (TIF) [file pone.0210877.s001.tif]
